# Smelling Wellness: Associations Between Botanic Garden Scentscapes and Human Health Gains

**DOI:** 10.3390/ijerph23030304

**Published:** 2026-02-28

**Authors:** Molly Rose Tucker, William Kay, Kieran Storer, Anya Lindström Battle, Katherine Willis

**Affiliations:** Department of Biology, University of Oxford, Oxford OX1 3EL, UK; william.kay@biology.ox.ac.uk (W.K.); kieran.storer@biology.ox.ac.uk (K.S.); anya.lindstrombattle@gmail.com (A.L.B.); kathy.willis@biology.ox.ac.uk (K.W.)

**Keywords:** anxiety, biogenic volatile organic compounds, health, monoterpenes, physiological indices

## Abstract

**Highlights:**

**Public health relevance—How does this work relate to a public health issue?**

- Given the increasing prevalence of stress-related health disorders and the continued urbanisation of populations, there is an increasing need for accessible nature-based interventions in urban environments.
- Accordingly, this study examines how the plant-based scent profiles found in botanic glasshouses could contribute to measurable changes in health and wellbeing outcomes.

**Public health significance—Why is this work significant to public health?**

- Observations of reduced anxiety, negative emotions and heart-beat rate after 30 min glasshouse exposure are consistent with the previous findings from clinical studies on the health effects of scents released by plants.
- The identification of distinct plant scent profiles previously associated with therapeutic effects in clinical settings, highlights the potential for vegetation-rich environments to deliver meaningful physiological and psychological health benefits.

**Public health implications—What are the key implications or messages for practitioners, policy makers and/or researchers in public health?**

- Differences in responses between the glasshouses suggest that the airborne compounds released by certain plant species may be associated with psychological and physiological calming.
- These results reinforce the importance of considering smell (scentscapes) along with sight and sound in urban green space planning and policy.

**Abstract:**

This pilot study investigated whether ambient biogenic volatile organic compounds (bVOCs)—scent profiles emitted by botanic glasshouse vegetation—could contribute to quantifiable human health and wellbeing outcomes. Over 11 months in 2024 (January–December), human participant trials were conducted at the Oxford Botanic Garden to compare the physiological and psychological effects associated with spending 30 min exposures in five different vegetation-rich glasshouses, each characterised by a distinct and complex bVOCs profile, with those of a plant-free room. Pre- and post-intervention assessments were conducted on 43 participants, using the State-Trait Anxiety Inventory (STAI), heart-beat rate (beats per minute), and heart rate variability (HRV): the latter two are widely used as an index of regulation of the autonomic nervous system. Significant reductions in STAI anxiety scores and decreases in heart-beat rate were observed, while HRV indices remained stable, relative to the plant-free room, following glasshouse exposure. Distinct scent profiles in the glasshouses included compounds that have previously shown associations with therapeutic effects in clinical settings, indicating the potential of these scented vegetation-rich glasshouse environments to promote the beneficial health effects observed in this study. Overall, these findings highlight the potential public health value of aromatic plant species and the importance of incorporating them into urban green space planning and policy.

## 1. Introduction

Biogenic volatile organic compounds (bVOCs) are molecules synthesised and released by plants, animals, and fungi, characterised by high vapour pressures and low water solubilities. The release of bVOCs from plants can be triggered by a range of abiotic and biotic factors [1,2,3] and are associated with aspects of plant communication such as attracting pollinators and deterring herbivores [3,4]. The distinct scent profiles of different green spaces arise from the unique combination of bVOCs emitted by the present plant species, alongside seasonal variation and plant health status.

Emerging clinical evidence suggests that bVOCs can also have significant effects on human health [1,5]. Inhalation of specific plant odours can trigger psychological and physiological responses via both olfactory and systemic pathways [1,6]. In the olfactory pathway, scent molecules enter the nasal cavity and bind to receptors in the olfactory epithelium, initiating neural signals that are relayed via the olfactory bulb to brain regions involved in emotional regulation, cognitive function, and autonomic stress responses [6,7,8]. In the systemic pathway, small plant-derived molecules such as terpenes enter the bloodstream via the lungs, where they may cross the blood–brain barrier or interact with peripheral receptors to induce physiological changes associated with involuntary neural and endocrine pathways, including alterations in heart-beat rate, heart rate variability, and circulating stress hormones such as adrenaline and cortisol [1,6,7,9].

Exposure to forested environments, where these bVOCs are present in the ambient air, has also been associated with physiological and psychological responses that are similar to those observed in clinical inhalation trials [1,5,10,11,12,13,14,15,16,17]. These effects are thought to arise, at least in part, from inhalation of plant odours in the forest air, engaging both the olfactory and systemic pathways described above. However, in natural settings, additional sensory inputs from the visual and auditory characteristics of nature also contribute to these outcomes, as they are independently known to elicit comparable responses [9,18,19,20,21,22,23,24,25,26]. Reported changes include reductions in perceived stress and anxiety, improvements in mood and attention, enhancements in subjective restoration, and measurable shifts in physiological markers such as lowered blood pressure, reduced heart-beat rate, increased heart rate variability, and decreased circulating cortisol levels [5,10,11,12,13,15,17,27,28,29,30].

Other studies have demonstrated enhanced natural killer (NK) cell activity in participants’ blood following walks in coniferous forests, with important implications for human health because NK cells are important for immune defence against viruses and cancer [27,28,30,31]. While the precise mechanisms underlying these effects are still under investigation, the preliminary evidence points to a role for α-pinene in forest air. In vitro experiments show that α-pinene can directly enhance NK-cell activation and cytotoxicity via the ERK and AKT signalling pathways (two major intracellular cascades that regulate NK-cell growth, survival, and activation) [32]. Taken together with observations that time spent walking in pine forests leads to elevated α-pinene concentrations in participants’ blood, these findings suggest that circulating α-pinene may act as an internal switch that boosts NK-cell activity and cancer-cell killing [32].

This increasing body of data on the health benefits of spending time in nature has, in recent years, contributed to the emergence of ‘green prescriptions’: namely, the use of exposure to nature to promote mental and physical wellbeing [33]. However, to date, research exploring the specific effects of plant bVOCs on human health outcomes have primarily been conducted in clinical settings or forested ecosystems [1,16]. As a consequence, the impacts of being exposed to ambient bVOCs, and their links to human health outcomes in urban green spaces such as parks and botanic gardens, where people are most likely to encounter them, have been relatively poorly studied [16,24]. This raises important questions including: (i) Do the beneficial effects of inhaling bVOCs seen in clinical and forest settings also occur when encountering bVOCs in ambient urban green spaces? (ii) Do human physiological and psychological responses vary between urban green spaces which contain different scents due to their composition of plant species?

To begin to explore these questions, our pilot study examined whether physiological and psychological outcomes—similar to those reported in forest and clinical settings, and attributed to olfactory and systemic pathway stimulation—could be detected when participants inhaled bVOCs present in the ambient air in five glasshouses in Oxford Botanic Garden (OBG) compared to a plant-free room. In particular, we aimed to test whether exposure resulted in the modulation of autonomic activity (heart-beat rate and heart rate variability) and psychological responses such as reduced anxiety [8]. The purpose of this pilot study was therefore to assess whether comparable health outcomes could be observed; we did not attempt to determine the specific mechanisms of action involved, nor did we investigate the potential pharmacological effects, such as NK cell activation.

Specifically, our objectives were to (i) characterise the profile of ambient bVOCs and calculate the amount of green foliage present across the five OBG glasshouses and a plant-free room and (ii) analyse any associated physiological and psychological effects in participants who spent 30 min sitting in each glasshouse and the plant-free room. This study took place over 11 months, between January–December 2024.

## 2. Materials and Methods

### 2.1. Study Site

**Figure 1:**
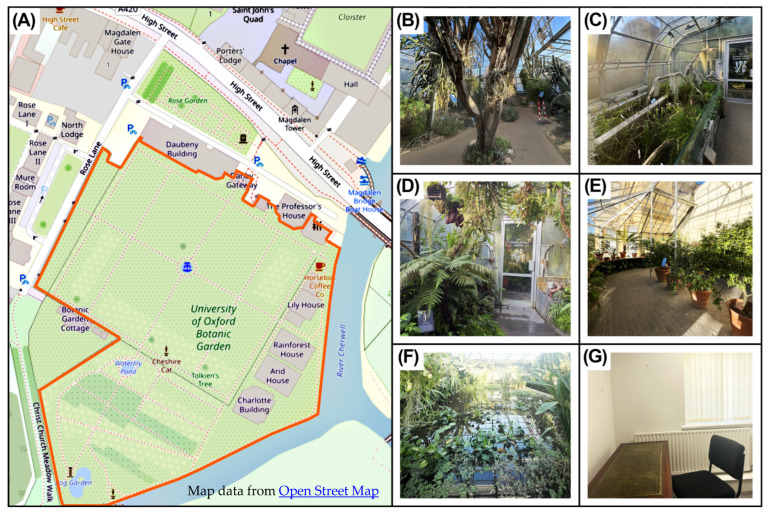
(**A**) Open street map of the Oxford Botanic Gardens (OBG), with the boundaries of the garden highlighted in red [37]. The site contains publicly accessible glasshouses, each offering distinct plant collections. (**B**–**G**) OBG glasshouses included in the study: (**B**) Arid, (**C**) Carnivorous Plant, (**D**) Cloud Forest, (**E**) Conservatory, (**F**) Waterlily, and (**G**) plant-free room.

The study was conducted in five glasshouses at the Oxford Botanic Garden (OBG)—Arid, Carnivorous Plant, Conservatory, Cloud Forest and Waterlily—as well as a plant-free room (Figure 1). This study aimed to emulate a realistic experience of time spent in an indoor room compared with an urban green space, and accordingly, this plant-free setting served as a minimal-stimulation comparator, rather than a strict control [16,24,34]. The bVOCs profile of each of the glasshouses was measured during three periods to capture seasonal variation: January–February 2024, June–August 2024 and November–December 2024. Using the Oxford Botanic glasshouses enabled us to account for all plant species present and their abundance [35], as well as minimising any effects from natural and unnatural auditory stimulation, such as bird song, rustling of leaves and traffic sounds [23,26,36].

### 2.2. Mapping Ambient bVOC Profiles

To measure the ambient bVOC profiles in each of the glasshouses and the plant-free room, air samples were simultaneously captured in four Markes Tenax TA tubes during a sampling event which lasted for at least 30 min. We used a GilAir^®^ pump with a flow rate of 400 mL/min (100 mL/min/tube). The tubes were located at a height of ~1.5 m to ensure that we captured the bVOCs at a vertical level in the ambient air closest to that experienced by the human nose (adapted from Walker et al. 2023) [38]. Using these indoor urban spaces also allowed us to map the bVOC profiles in the climate-controlled glasshouses, therefore minimising variations in temperature and humidity, which are known to impact bVOC composition [1,4,38,39]. On average, the temperatures and humidity ranged between 15 and 22 °C and 47 and 75%, respectively.

To account for the diurnal variability of bVOC release, bVOC samples were taken during three sampling events between 8 a.m. and 2 p.m. for each glasshouse [38]. The bVOC compounds in the air samples were analysed using gas chromatography–mass spectrometry (GC-MS), following standard procedures adapted from Kay et al. (2026) [40]. Chromatographic output data were processed using AMDIS software (v2.73) [41], which enabled the identification of compounds by combining spectral library identification (NIST20) and retention index (RI) matching. Deconvolution settings used high sensitivity and medium shape requirements to separate overlapping compound peaks. Peak areas from blank control tubes were subtracted to account for compounds originating from Tenax filters, TD trap, or GC column.

### 2.3. Volatile Data Analysis

The chemical profiles obtained from GC–MS were analysed using multivariate statistics. For principal component analysis (PCA) and partial least squares discriminant analysis (PLS-DA), abundances were standardised within each sample (row standardisation) and then square-root transformed. This ensured that differences between samples reflect overall patterns in composition, rather than large differences in total abundance. The PCA was carried out using the FactoMineR package in R [42], and visualised with factoextra [43].

To further distinguish between volatiles, we characterised compounds into whether they were primarily biogenic or anthropogenic. The average amounts of all identified bVOCs were summed and compared across six sampling sites, as well as those compounds found uniquely at a single sampling site. To explore how many potentially human-health-promoting volatiles were identified in each site, we used a presence/absence heatmap visualisation of monoterpenes, sesquiterpenes, and terpenoids.

To assess pollution in the sampling sites, we assessed the relative amounts of benzene, toluene, ethylbenzene, and xylene (p, m, and o-xylene)—collectively named BTEX compounds—which are well-documented sources of anthropogenic pollution [44]. Thirdly, as a metric for healthy/unhealthy compounds, we used the ratio of the summed abundance of bVOCs to BTEX compounds (∑bVOCs/∑BTEX) [45].

All plots were produced in R, using ggplot2 [46], with colours manually selected from the RColorBrewer “Dark2” palette for consistency and clarity [47].

### 2.4. Mapping Ambient Foliage Greenness

Given the multisensory nature of the glasshouse environments and the previous literature demonstrating beneficial associations between vegetation and human health outcomes [18,19,20,21], visual stimulation from the botanical plants was also considered a potential contributing factor to the observed physiological and psychological responses. To account for the relative amount of green foliage within each of the glasshouse environments, digital colour images were taken of each glasshouse and the density of green foliage was measured using ImageJ (Version 1.54g) processing software to calculate the percentage area of green coverage across 180°—taken to represent a participant’s field of vision [48] (See Supplementary Materials).

### 2.5. Participants and Study Design

A total of 43 participants (33 females; 10 males) aged 18 and over were recruited in three rounds between 18th January and 3rd December 2024. The experiments were conducted over three periods, from (i) January–February 2024, (ii) June–August 2024 and (iii) November–December 2024. Participants were students at the University of Oxford or volunteers associated with the Oxford University Gardens, Libraries and Museums. Full ethical approval was granted for the study before its commencement (Central University Research Ethics Committee (CUREC) reference R89114/RE001/RE002). After providing the participants with the study objectives and requirements, written consent was obtained in advance of commencing the research.

Before glasshouse exposure, psychological and physiological measurements—STAI, heart-beat rate (bpm) and HRV indices (SDNN)—were taken in the plant-free room. Participants were then randomly allocated to one of the five glasshouses or the plant-free room. Participants spent 30 min seated alone in their intervention location and were instructed to refrain from any physical activity. After the intervention, the same set of physiological and psychological measurements were recorded for participants. Participants returned weekly and were assigned a new intervention each week semi-randomly, so that over the trial, each participant was exposed to each glasshouse, and to ensure there were no repeated sessions in the same glasshouse.

### 2.6. Physiological and Psychological Markers

To discern psychological anxiety, the participants filled in the State-Trait Anxiety Inventory (STAI) questionnaire before and after the 30 min (See Supplementary Materials). This inventory, which involves the participants answering twenty questions, is a well-established psychological method [16,49,50] where the questions have been devised to measure both state and trait anxiety that is not specific to any psychiatric disorder [16,49]. Once completed, the prior and post-glasshouse exposure STAI results for each participant were coded and assigned a predetermined score, with values between 20 and 80. Higher STAI scores have previously been shown to be indicative of more severe anxiety and negative emotions [51].

Heart-beat rate (bpm) is widely recognised as a physiological indicator of stress, with increased rates often being associated with elevated levels of anxiety [52]. Heart-beat rate (bpm) is regulated by the autonomic nervous system, with the parasympathetic nervous system suppressing activity and the sympathetic nervous system increasing beats per minute in stressful situations [52]. To measure heart-beat rates (bpm), participants were provided with user-friendly technology including Fitbit Inspire 2 models and smartphones. The Welltory app (Version 4.26.0) was installed on all smartphones that used photoplethysmography (PPG) to measure heart-beat rate (bpm) and utilised the camera and flash to detect changes in blood volume in the user’s vessels [53,54]. Participants again recorded their own measurements before and after the session.

To monitor autonomic nervous activity, SDNN measurements—which represent a measure of heart rate variability (HRV), measured as the standard deviation of the NN interval and the quantification of the variation in time intervals between consecutive normal heart beats—were recorded using photoplethysmography (PPG) [55]. Both sympathetic and parasympathetic nervous system activity contribute to SDNN [56]. However, in short-term recordings, SDNN is primarily a measure of parasympathetic activity, where higher values are typically indicative of increased physiological relaxation [15,57,58]. To measure SDNN, the participants used the Welltory app (Version 4.26.0) installed on a smartphone and recorded measurements before and after the experiment [54].

### 2.7. Human Health Data Analysis

Microsoft Excel was used to organise participant data, and R was used for statistical analyses using R studio (version 2025.05.0+496) [59]. To compare STAI scores, heart-beat rate and HRV before and after the exposure, paired Wilcoxon signed-rank tests were performed to assess whether post-exposure values were significantly lower than pre-exposure values (alternative = After < Before). Then, per-site sample sizes and test statistics were recorded, and significance levels were annotated on boxplots. Finally, to compare STAI, heart-beat rate and HRV results across different sites, linear mixed-effects models were fitted with change (Δ) values with the response and glasshouse as the fixed effect, including participant experiment as a random intercept to account for repeated measures. % Change (Δ) values for each of the STAI scores, heart-beat rate and HRV were calculated using the following formula: ((After value − Before value)/Before Value) * 100.

Models were estimated using the lmerTest package [60], with type-III ANOVAs used to assess the overall effect of the glasshouse. Post hoc comparisons (plant-free room vs. each glasshouse) were performed using emmeans [61] with Benjamini–Hochberg correction. Tukey’s HSD contrasts across all sites were summarised as compact letter displays, using the *multcompView* package in R [62]. For select models, baseline (Before) values were added as covariates to control for starting differences, and conditional/marginal R^2^ values were calculated with *performance* [63] and plotted as boxplots.

To examine whether greenness also had an effect on the health outcomes, Spearman’s correlation test was performed to examine the relationship between the measured changes in physiological and psychological responses after participants had spent 30 min in the glasshouses and the amount of green foliage present in each of the glasshouses, using the *stats* package in R [59].

## 3. Results

### 3.1. Volatile Profiles Differ Between Glasshouses and Plant-Free Room

**Figure 2:**
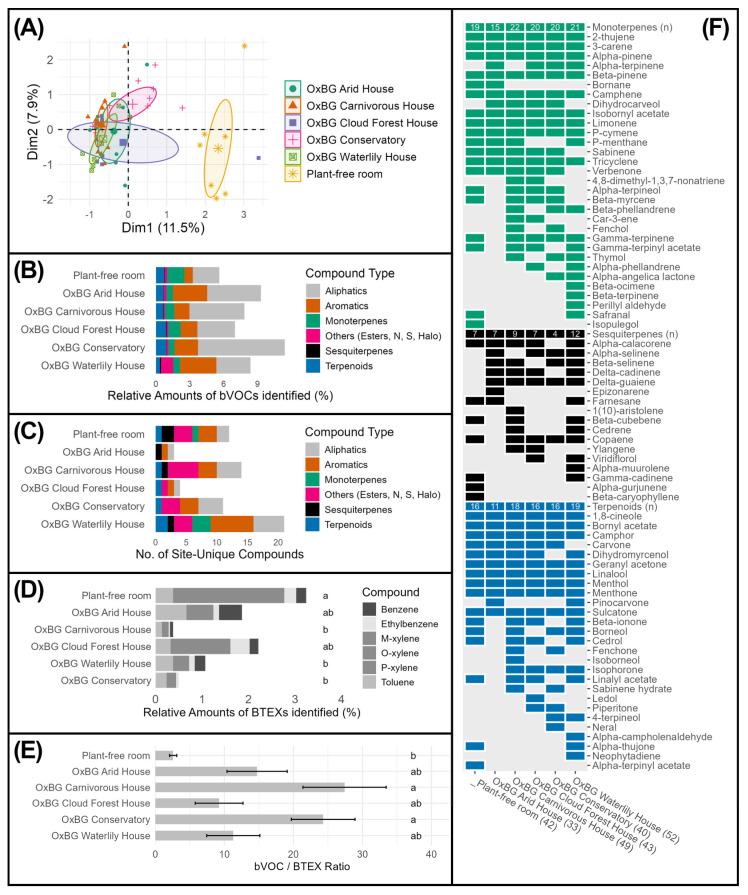
Multivariate patterns and compositional differences in volatile organic compounds across Oxford Botanic Garden glasshouses and the plant-free room. (**A**) Principal component analysis (PCA) of volatile profiles from all samples, following Hellinger transformation, with points coloured by the sampling environment and 95% confidence ellipses shown for each group. Percentages on axes indicate variance explained. (**B**) Relative contributions of major compound classes (aliphatics, aromatics, monoterpenes, sesquiterpenes, terpenoids, and others) to total identified biogenic volatile organic compound (bVOC) abundance within each environment. (**C**) Number of site-unique compounds detected at each location, grouped by compound class. (**D**) Relative proportion of BTEX compounds identified at each site, with letters indicating significant differences among locations (one-way ANOVA with Tukey post hoc tests, *p* < 0.05). (**E**) Ratios of total bVOC to BTEX abundance across locations (mean ± SE), with higher values indicating greater dominance of bVOCs. Letters denote statistical groupings (Tukey post hoc tests). (**F**) Presence–absence matrix of terpene and terpenoid compounds across sampling environments, grouped by compound class. Filled cells indicate detection of a compound at a given site, with totals reported beneath each location for major terpene classes.

An initial PCA indicated significant separation between volatile profiles from the plant-free room and the glasshouse environments (Figure 2A, PERMANOVA (F_5,46_ = 1.80, R^2^ = 0.164, *p* = 0.019)). All glasshouse sites exhibited higher total bVOC abundances compared to the plant-free room; however, this difference was not statistically significant (one-way ANOVA, *p* = 0.17; Figure 2B). A further assessment of compounds found uniquely at a single sampling site found that the Waterlily House was the richest, with 21 unique bVOCs (Figure 2C). All other sites ranged from 3 to 14 unique compounds.

The proportion of BTEX compounds was highest in the plant-free room (2.88%; Figure 2D). Three glasshouse environments showed significantly lower BTEX proportions (ANOVA with Tukey post hoc tests, *p* < 0.05), ranging from 0.85% in the Waterlily House to 0.33% in the Carnivorous Plant House. These differences were primarily driven by a substantially higher proportion of xylene isomers (p-, m-, and o-xylene) in the plant-free room, relative to the glasshouses.

To summarise the biogenic, relative to the anthropogenic contributions, the bVOC:BTEX ratios were calculated (Figure 2E). The Carnivorous Plant House showed the highest ratio (27.4:1), followed by the Conservatory (24.3:1), reflecting strong biogenic dominance. Although all glasshouses showed higher ratios than the plant-free room, these differences were not statistically significant (Tukey post hoc tests, *p* > 0.05).

Across the three volatile sampling seasons, a combined total of 79 terpenes and terpenoids were identified (Figure 2F). The Waterlily House exhibited the greatest diversity, with 51 compounds detected, while the Arid House contained the fewest (33). Of the total terpene and terpenoid pool, 34 compounds were not detected in the plant-free room. When compounds were grouped by class, the Waterlily House contained the highest numbers of both sesquiterpenes (14) and terpenoids (19) of all sampling locations, including the plant-free room. It also exhibited the second-highest number of monoterpenes (20), slightly lower than the Carnivorous Plant House, which contained the greatest monoterpene diversity (22).

### 3.2. Significant Benefits to Psychological Wellbeing Were Observed Following Time Spent in All Glasshouses

**Figure 3:**
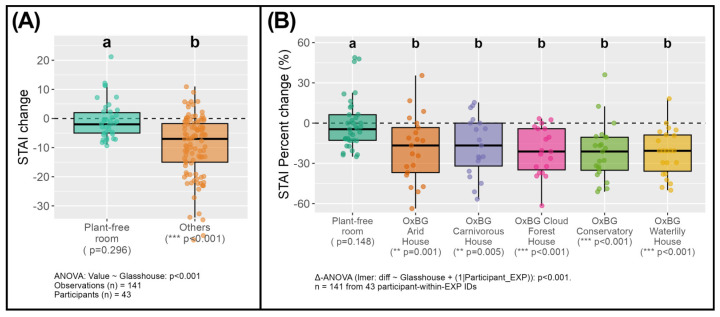
Changes in anxiety (State-Trait Anxiety Inventory (STAI)) scores, following exposure to Oxford Botanic Garden glasshouses and a plant-free room. (**A**) Percentage change in STAI scores for the plant-free room compared with all glasshouse environments combined. Points indicate individual observations; boxplots show medians, interquartile ranges, and 1.5 × IQR whiskers. Different letters denote significant differences between groups based on a one-way ANOVA with Tukey post hoc tests (*p* < 0.05). The horizontal dashed line indicates no change from baseline. (**B**) Percentage change in STAI scores across individual sampling environments. Points represent repeated observations across participants and experiments; boxplots show medians, interquartile ranges, and 1.5 × IQR whiskers. Letters indicate significant differences among environments, based on a mixed-effects delta-ANOVA with participant-within-experiment included as a random effect and Tukey-adjusted post hoc comparisons (*p* < 0.05). Within-site *p*-values displayed beneath x-axis labels correspond to paired Wilcoxon tests comparing pre- and post-exposure scores. Labels ** and *** below the x-axis indicate statistical significance at *p* < 0.01 and *p* < 0.001, respectively. The horizontal dashed line indicates no change from baseline.

Across 141 observations from 43 participant-within-experiment IDs, changes in anxiety scores were analysed following exposure to different indoor environments. When environments were grouped into the plant-free room versus all glasshouse locations, a significant difference in percentage change in STAI scores was detected (one-way ANOVA, *p* < 0.001; Figure 3A). Glasshouse environments were associated with lower anxiety change scores, whereas no significant change was observed in the plant-free room.

When individual sampling environments were considered separately, a mixed-effects delta-ANOVA revealed the significant effect of the site on percentage change in STAI scores (F_5,114.8_ = 5.49, *p* < 0.001; Figure 3B). All glasshouse environments were associated with reductions in anxiety scores relative to the baseline, whereas the plant-free room showed no significant change. Pairwise post hoc comparisons indicated that the anxiety reductions observed in each glasshouse environment were greater than those in the plant-free room (Tukey-adjusted *p* < 0.05), while differences among glasshouse environments were more variable.

### 3.3. Physiological Response (Heart-Beat Rate and Heart Rate Variability) Indicated Glasshouse Specific Reductions

**Figure 4:**
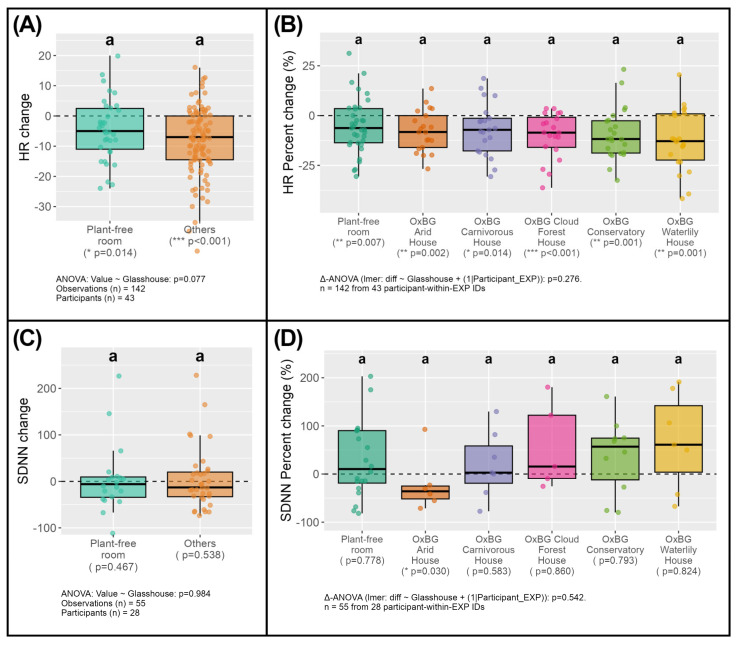
Heart-beat rate (HR) and heart rate variability (SDNN) responses following exposure to Oxford Botanic Garden glasshouses and a plant-free room. (**A**) Change in HR, comparing the plant-free room with all glasshouse environments combined. (**B**) Percentage change in HR across individual sampling environments. (**C**) Change in SDNN, comparing the plant-free room with all glasshouse environments combined. (**D**) Percentage change in SDNN across individual sampling environments. Points represent individual observations; boxplots show medians, interquartile ranges, and 1.5 × IQR whiskers. Letters denote groupings based on one-way ANOVA (**A**,**C**) or mixed-effects delta-ANOVA (**B**,**D**) with participant-within-experiment included as a random effect and Tukey-adjusted post hoc comparisons. Within-site *p*-values shown beneath x-axis labels correspond to paired Wilcoxon tests comparing pre- and post-exposure values. Labels *, ** and *** below the x-axis indicate statistical significance at *p* < 0.05, *p* < 0.01 and *p* < 0.001, respectively. Dashed horizontal lines indicate no change from baseline.

Within-site paired comparisons revealed significant reductions in heart-beat rate following exposure in the Arid, Cloud Forest, Conservatory, and Waterlily Houses (paired Wilcoxon tests, *p* < 0.05; Figure 4B), suggesting site-specific reductions in heart-beat rate compared to time spent in the plant-free room, and Carnivorous House. However, given that modest reductions in heart-beat rate after 30 min also occurred in the plant-free and Arid room, when the grouped heart-beat rate reductions were compared to the plant-free room, the overall effect of HR change scores did not indicate a significant difference in heart-beat rate reduction between the plant-free room and all other glasshouses (one-way ANOVA, *p* = 0.077; Figure 4A).

For HRV, SDNN change scores showed substantial inter-individual variability, although elevated parasympathetic activity is apparent in the Cloud Forest, Conservatory and Waterlily Houses—however, these are not statically significant when comparing among individual sites (mixed-effects delta-ANOVA, *p* = 0.542; Figure 4D). In addition, no statistically significant differences were detected between the plant-free room and glasshouse environments when grouped (one-way ANOVA, *p* = 0.984; Figure 4C). It must also be noted, however, that due to measurement errors, the sample sizes for this metric were small and this almost certainly limited the power of the analysis.

### 3.4. Visual Stimulation from Glasshouse Foliage May Contribute to Wellbeing Benefits

**Figure 5:**
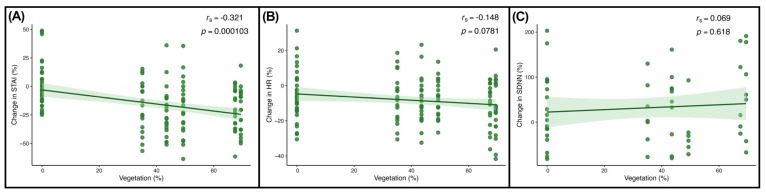
Associations between vegetation cover and physiological and psychological responses. Scatter plots show the percentage change in (**A**) STAI scores, (**B**) heart-beat rate (HR), and (**C**) heart rate variability (SDNN) in relation to the percentage of vegetation cover present in the glasshouses and the plant-free room. Points represent individual observations, and the green shaded area indicates the 95% confidence interval. Spearman’s rank correlation coefficient (r_s_) and the corresponding *p*-values are indicated in the top-right hand corner.

In addition to the smell of foliage, we tested for associations from seeing vegetation in each of the glasshouses and the plant-free room, and the measured physiological and psychological responses (Figure 5A). A small but marked negative trend was found between the amount of green foliage present and the percentage change in the STAI scores among participants (*r_s_* = −0.321, *p* = 0.000103). However, no significant associations were observed between HR or SDNN and the amount of green foliage (HR: *r_s_* = −0.148, *p* = 0.0781; SDNN: *r_s_* = 0.069, *p* = 0.618) (Figure 5B,C).

## 4. Discussion

### 4.1. Profiling the Scentscapes in the OBG Glasshouses and Plant-Free Room

Our measurements confirmed that the five Oxford Botanic Garden glasshouses were characterised by distinct ambient bVOC profiles compared with the plant-free room, indicating that each glasshouse constituted a chemically unique scentscape (Figure 2A). The differences in volatile composition are almost certainly related to the contrasting plant assemblages maintained in each environment, consistent with previous studies showing that species identity and plant functional type strongly shape emitted bVOC blends [35]. Many of the bVOCs detected in the glasshouses, including several monoterpenes and terpenes, have been associated with beneficial psychological or physiological effects in clinical studies, providing a clear rationale for examining their potential association to wellbeing outcomes in these settings (Figure 2F, Table 1) [35].

Within this overall pattern, the Waterlily glasshouse was notable for exhibiting the highest number of unique biogenic compounds, relative to the other glasshouse environments (Figure 2C,F). This richer and more distinctive bVOC profile suggests that visitors to this space are exposed to a particularly complex olfactory environment, which may have implications for the range of bioactive molecules available for inhalation. The marked separation of bVOC signatures among glasshouses implies that participants encountered a suite of chemically diverse, plant-driven microhabitats, rather than a homogeneous “generic” plant odour.

The plant-free room, while statistically distinct from the glasshouses in its overall volatile profile, still contained a small number of terpenes, including α-pinene, β-caryophyllene, camphene, limonene and 1,8-cineole (Figure 2F) [64,65,66,67,68,69]. However, the plant-free room was also characterised by relatively higher abundances of BTEX compounds (benzene, toluene, ethylbenzene and xylenes), which have been recognised as detrimental to human health, including respiratory and neurological impacts, and are typical of urban pollution sources (Figure 2D,E) [44,70]. Together, these findings highlight a sharp contrast between the chemically complex, plant-enriched scentscapes of the glasshouses and a more pollution-skewed profile in the plant-free indoor environment. This contrast provides important context for interpreting the associated physiological and psychological responses found in the glasshouses compared to the plant-free room and is described in the subsequent sections.

**Table 1:** Plant bVOCs detected in OBG glasshouses and specific health benefits identified in previous studies.

Volatile	Detected In	Associated Health Effects
α-pinene	Arid, Carnivorous, Cloud Forest, Conservatory, Waterlily, Plant-free room	Antidepressant effects, reductions in anxiety and increased parasympathetic activity [1,69,71,72]
β-myrcene	Carnivorous, Cloud Forest, Conservatory, Plant-free room	Anti-anxiety effects [73,74,75]
ß-caryophyllene	Waterlily	Anti-anxiety effects [76,77]
Bornyl acetate	Arid, Carnivorous, Cloud Forest, Conservatory, Waterlily, Plant-free room	Autonomic relaxation effects such as altered HRV [78,79]
Camphene	Arid, Carnivorous, Cloud Forest, Conservatory, Waterlily, Plant-free room	Therapeutic health effects, including cardiovascular protection [68]
Camphor	Arid, Carnivorous, Cloud Forest, Conservatory, Waterlily, Plant-free room	Psychophysiological relaxation, including decreased heart-beat rate and altered brain-wave activity [80]
Cedrol	Carnivorous Plant, Cloud Forest, Waterlily	Autonomic relaxation, including changes in HRV [81]
Limonene	Arid, Carnivorous, Cloud Forest, Conservatory, Waterlily, Plant-free room	Reduced anxiety and physiological relaxation, including decreased heart-beat rates [64,66,73]
Linalool	Arid, Cloud Forest, Conservatory, Plant-free room	Antidepressant activity [82,83,84]
Menthol	Arid, Carnivorous, Cloud Forest, Conservatory, Waterlily, Plant-free room	Physiological effects, including changes in immune response and reduced pain [85]
Neophytadiene	Waterlily	Neuroprotective effects [86]
Perillaldehyde	Waterlily	Anti-depressant effects [87]
γ-terpinene	Carnivorous, Cloud Forest, Conservatory, Waterlily	Relaxation responses, such alterations in brain-wave activity [11,66]
1,8-cineole	Arid, Carnivorous, Cloud Forest, Conservatory, Waterlily, Plant-free room	Reductions in anxiety and modified physiological effects [65,88]

### 4.2. Psychological and Physiological Responses Associated with Time Spent in Different OBG Glasshouses

Given that many of the bVOCs identified in these glasshouses have previously been shown to affect physiological and psychological changes, our second aim was to ask whether spending time in these environments was associated with measurable changes in participants’ wellbeing.

In terms of psychological changes, our findings indicated that reductions in anxiety and the alleviation of negative emotions, as recorded in the STAI scores, were associated with 30 min in each of the glasshouses, compared with the plant-free room (Figure 3A,B). This pattern is consistent with reports that exposure to plant scents in forested environments is associated with improved mood [1,5,11,17] and clinical studies in which the inhalation of plant-emitted scents triggered mechanisms of action in the olfactory and systemic pathways, as discussed in the introduction, leading to psychological calming [64,71,74,81,88,89].

Within the different glasshouses, larger reductions in anxiety (STAI scores) were apparent following time spent in the Cloud Forest, Conservatory and Waterlily glasshouses (Figure 3B), suggesting that these scentscapes may be associated with stronger psychological benefits, potentially in part due to their distinctive and complex volatile profiles (Figure 2 and Figure 3) [6,64,69,71,78,80]. Previous work also indicates that site-specific bVOC signatures can be related to differences in mood outcomes [16,17]. For example, Donelli et al. (2023) [16] reported that exposure to high concentrations of sabinene and o-cymene had minimal influence on mood, whereas inhalation of α-pinene was associated with marked reductions in anxiety during forest-therapy sessions at different sites. Other studies have also shown that distinct floral scents can either increase or decrease anxiety-related brainwave activity [90,91], reinforcing the notion that scent quality and composition matter. An important area of future work will be to build upon this pilot study by quantifying absolute bVOC concentrations in each glasshouse to enable a more precise interpretation of any associations with positive psychological outcomes [1,16,92,93,94].

Despite also finding a number of terpenes linked with reduced anxiety or improved mood in the ambient air in the plant-free room (Figure 2E,F), we did not observe associated improvements in psychological wellbeing following time spent in this setting (Figure 3A,B). This difference may be attributable to the comparatively higher abundances of BTEX compounds, relative to plant-derived bVOCs (Figure 2B,E), in this plant-free room. Previous studies have shown that BTEX compounds, which are common urban pollutants, have the potential to cause irritation and neurotoxic effects at elevated concentrations, and even at low levels, they may influence mood or cognitive performance [44,70,95]. One possible interpretation, therefore, is that the presence of these anthropogenic compounds could have masked or counteracted the beneficial effects of the plant-derived volatiles, thereby weakening the restorative responses. Alternatively, the observed differences may reflect the broader multisensory context of the glasshouse environments [24]. As the study design prioritised ecological validity rather than isolating olfactory stimuli, participants were exposed simultaneously to the scents and visual characteristics of the vegetation in the glasshouses [24]. These combined sensory inputs may have moderated the psychological outcomes and contributed to the distinct patterns observed between glasshouses and the plant-free comparison room [18,19,20,21]. This interpretation is supported by the modest association that is apparent in our modelling between foliage cover and STAI scores detected across the glasshouses (Figure 5A).

Taken together, our findings extend previous work by indicating that, within the urban greenspace context of the OBG glasshouses, variation in the diversity and composition of plant-emitted volatiles, alongside other environmental stimuli, may be associated with differences in psychological responses. The results from this pilot study also suggest that different glasshouse environments may be differentially associated with these outcomes, underscoring the value of further research in this setting.

In terms of physiological changes, exposure to all glasshouse environments was associated with small reductions in heart-beat rate (bpm), suggesting mild physiological relaxation among participants. However, similar declines were also observed in the plant-free room (Figure 4A,B). The detection of volatiles such as α-pinene and limonene in the ambient air of the plant-free room (Figure 2F) may account for this effect, despite the absence of vegetation [64,71]. Parasympathetic heart rate responses also showed limited differentiation between the glasshouses and the plant-free room (Figure 4A,B), but these were not statistically significant, although some indications of elevated parasympathetic activity were apparent in certain glasshouses (Figure 4C,D).

These results partly diverge from earlier studies that reported more pronounced associated cardiovascular responses to inhaled plant volatiles [71,80,96,97,98] and combined visual–olfactory exposure to greenery [21]. However, the modest sample size in this pilot study likely constrained statistical power to detect subtle physiological effects [99,100], and the weak HRV responses may reflect measurement limitations, rather than a genuine absence of physiological changes [24,53,93,101].

With appropriate caution, the limited cardiovascular evidence suggests that time spent in the Cloud Forest, Conservatory, and Waterlily glasshouses may modestly reduce heart-beat rate and enhance parasympathetic activity, relative to other settings. Future research should employ broader physiological indicators, such as blood pressure and cortisol, and larger participant cohorts, to increase sensitivity to differential responses and clarify the contribution of bVOC inhalation to human health outcomes [6,24].

### 4.3. Limitations of the Study

The present study prioritised ecological validity by employing a plant-free comparison room, rather than a strict laboratory control [24]. While this design provided a realistic evaluation of human responses to garden environments, it consequently limited the capacity to disentangle the effects of ambient bVOCs from other sensory stimuli that are inherent to the glasshouse settings [24]. It is therefore possible that environmental variables such as light intensity, temperature, humidity, and visual factors contributed to some of the observed health responses. Future research could address this limitation through refined methodological approaches: for instance, by using a filtering mask that is capable of removing ambient bVOCs while preserving the multisensory experience of the glasshouse environment [93,102]. Incorporating a formal control group would further enhance experimental rigour and allow for a more precise attribution of outcomes to the presence of ambient volatiles [1,9].

Another limitation of this study is the absence of absolute quantification of volatile compound concentrations. Without absolute concentrations it was not possible to calculate the dosage or exposure intensity experienced by participants [16]. This pilot work aimed to explore the preliminary associations between environmental scent and human health responses, however, subsequent studies should integrate the quantification of airborne volatile concentrations and assess potential dose–response relationships [1,16,90]. Such data would facilitate comparison with controlled clinical and exposure studies, thereby strengthening inferences regarding causality. Finally, the small sample size and limited demographic diversity constrain the generalisability of the findings. Future investigations should recruit larger, more representative cohorts to enhance the statistical power and applicability of results to wider populations [97,99,103].

## 5. Conclusions

Our pilot study demonstrates that the Oxford Botanic Garden (OBG) glasshouses possess distinct and compositionally diverse bVOC profiles, with many compounds present that are known to contribute to positive physiological and psychological effects. The detection of key aromatic terpenes such as α-pinene, 1,8-cineole, linalool, and limonene across all glasshouses supports the potential of the bVOC profiles within these urban spaces to promote improvements in human health and wellbeing. In particular, the Cloud Forest, Conservatory, and Waterlily glasshouses exhibited the greatest diversity and abundance of biogenic volatile organic compounds that were previously demonstrated to enhance health outcomes, aligning with the observed associated improvements in participants’ reported decreases in anxiety and negative emotions following glasshouse exposure.

Our findings suggest that the specific composition and richness of natural scent blends, rather than the presence of individual compounds, may be a contributing factor to the restorative psychological benefits experienced in these biodiverse plant environments.

While physiological responses, such as heart-beat rate and parasympathetic activity, were less pronounced after 30 min in the glasshouses, modest declines in heart-beat rate hint at relaxation effects that likely warrant further investigation. The limited sample size and short exposure duration may have constrained detectable physiological differences. Nonetheless, the collective evidence indicates an association between plant-emitted volatiles and beneficial human wellbeing outcomes within these urban green space settings. Future studies incorporating larger cohorts, extended exposure times, additional physiological indicators, and stricter sensory isolation could clarify the mechanistic pathways linking bVOC exposure to health outcomes and help to refine the design of therapeutic plant-based environments in cities.

## Data Availability

All data are available by contacting the corresponding author.

## Supplementary Materials

The following supporting information can be downloaded at: https://www.mdpi.com/article/10.3390/ijerph23030304/s1. File S1: Supplementary Material for Smelling Wellness: Associations between Botanic Garden Scentscapes and Human Health Gains. Table S1. State-Trait-Anxiety-Inventory (STAI) used in this study as an indicator of psychological well-being. Figure S1. Examples of 180-degree images taken across the glasshouses from top to bottom: 1. Arid, 2. Carnivorous Plant, 3. Cloud Forest, 4. Conservatory and 5. Waterlily glasshouses.

## Abbreviations

The following abbreviations are used in this manuscript:

BTEX	Benzene; Toluene; Ethylbenzene; Xylene
bVOCs	Biogenic Volatile Organic Compounds
CUREC	Central University Research Ethics Committee
GC-MS	Gas Chromatography–Mass Spectrometry
HRV	Heart Rate Variability
OBG	Oxford Botanic Garden
STAI	State Trait Anxiety Inventory
PCA	Principle Component Analysis
PLS-DA	Partial Least Squares Discriminant Analysis
SDNN	Standard Deviation of NN intervals
