# Supplementary material for: Smelling Wellness: Associations Between Botanic Garden Scentscapes and Human Health Gains
Source: Int J Environ Res Public Health. 2026 Feb 28;23(3):304. doi: 10.3390/ijerph23030304 (PMC13026806; doi:10.3390/ijerph23030304)
Supplement: Supplementary file 1 [file ijerph-23-00304-s001.zip › ijerph-4113258-supplementary.pdf]

Supplementary Materials

Smelling Wellness: Associations between Botanic Garden Scentscapes and Human Health Gains

This supplement includes further details of the GC-MS methodology for identification and analysis of volatile organic compounds (VOCs) used within this study [1]. A copy of the State-Trait-Anxiety-Inventory (STAI) given to participants before and after glasshouse exposure (Table S1) is also contained within this supplement. Further contained in this supplement is the methodology followed in IMAGEJ to determine the relative vegetation coverage in each of the glass-houses (Figures S1).

The chromatographic output data was processed using the AMDIS software (v2.73), [2] which enabled identification of compounds by combined spectral and retention index (RI) matching, applying a minimum match factor of 70, an RI tolerance of +/- 10, and a strong match factor penalty. Deconvolution parameters were set to a component width of 25, adjacent peak subtraction of two, medium shape requirements and high sensitivity.

Compounds detected in fewer than half of technical replicates were removed from the dataset to exclude unreliable or inconsistent detections. Peak areas within technical replicates were normalised, firstly using the mean peak area of ethylbenzene, followed by calculating relative abundance (%) using the peak area of each compound divided by the total ion count within each sample tube (TIC).

<u>State-Trait-Anxiety-Inventory (STAI):</u>

Participants were asked to read each statement and select the most appropriate response to indicate how they felt right in the moment [3].

Table S1. State-Trait-Anxiety-Inventory (STAI) used in this study as an indicator of psychological well-being.

Statement	1.	Not at all	2.	A little	3.	Somewhat	4.	Very much so
1. I feel calm								
2. I feel secure								
3. I feel tense								
4. I feel strained								
5. I feel at ease								
6. I feel upset								
7. I am presently worrying over possible misfortunes								
8. I feel satisfied								
9. I feel frightened								
10. I feel uncomfortable								
11. I feel self- confident								
12. I feel nervous								
13. I feel jittery								
14. I feel indecisive								
15. I am relaxed								
16. I feel content								
17. I am worried								
18. I feel confused								
19. I feel steady								
20. I feel pleasant								

IMAGE J: Quantifying the vegetation coverage present across the glasshouses

Methodology

1. 180° degree panoramas were taken of each glasshouse for each iteration of the study

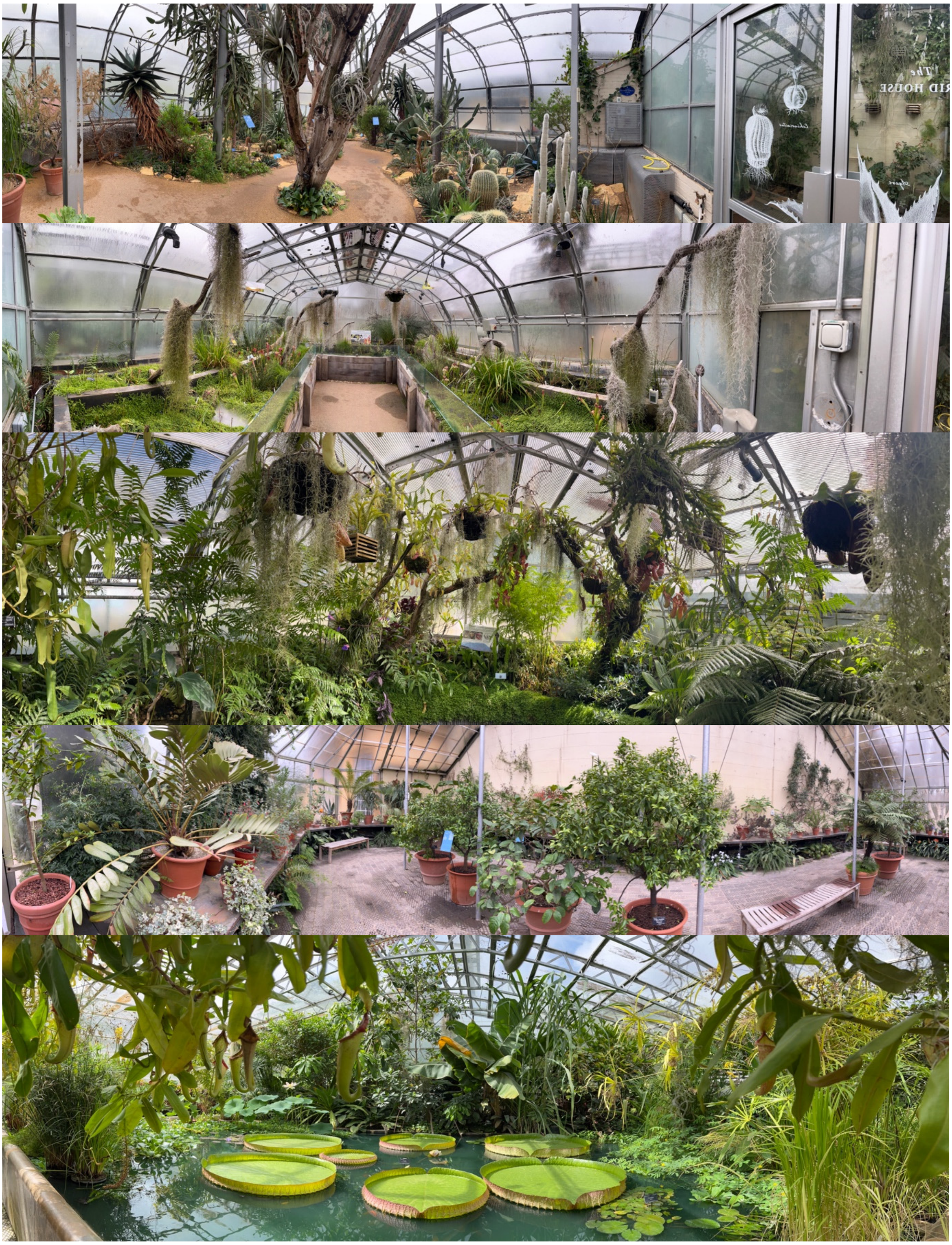

Figure S1. Examples of 180-degree images taken across the glasshouses from top to bottom: 1. Arid, 2. Carnivorous Plant, 3. Cloud Forest, 4. Conservatory and 5. Waterlily glasshouses.

2. In ImageJ [4], background interference, such as shades similar to that of vegetation was removed using the Freehand Selection tools. This included floor space, windows and roof where possible.
3. Th image was then saved as a pre-processed image.
4. The pre-processed image was then converted to 8-bit image.
5. The colour threshold of the image was then changed and set to LAB colour space.
6. Then LAB colour space values were adjusted until satisfied that the parts of the image (the vegetation present in each of the glasshouses) are selected for segmentation.

References

1. Kay, W.T.; Lindstrom Battle, A.L.; Humberstone, M.; Tucker, M.; Storer, K.; Kite, G.; Willis, K. A Walk in the Park - Identifying Healthy Greenspaces Using Scents. *medRxiv* **2026**, *submitted*.
2. National Institute of Standards and Technology. *Automated Mass Spectral Deconvolution and Identification System (AMDIS)*; National Institute of Standards and Technology: Gaithersburg, MD, USA 2023.
3. Spielberger, C. D. *State-Trait Anxiety Inventory for Adults (STAI-AD)*; APA PsycTests: Washington, DC, USA, 1983.
4. Schneider, C. A.; Rasband, W. S. NIH Image to ImageJ: 25 Years of Image Analysis. *Nat. Methods* **2012**, *9*, 671–675.
